# Phenolics Impart Au^3+^-Stress Tolerance to Cowpea by Generating Nanoparticles

**DOI:** 10.1371/journal.pone.0085242

**Published:** 2014-01-09

**Authors:** Nisha Shabnam, P. Pardha-Saradhi, P. Sharmila

**Affiliations:** 1 Department of Environmental Studies, University of Delhi, Delhi, Delhi, India; 2 Department of Chemistry, Indian Institute of Technology, New Delhi, Delhi, India; University of Kansas, United States of America

## Abstract

While evaluating impact of Au nanoparticles on seed germination and early seedling growth of cowpea, HAuCl_4_ was used as control. Seedlings of cowpea raised in HAuCl_4_, even at concentration as high as 1 mM, did not show any suppression in growth. Accordingly, Au^3+^, despite being a heavy metal, did not alter levels of stress markers (viz. proline and malondialdehyde) in cowpea. Interestingly, cowpea turned clear pale yellow HAuCl_4_ solutions colloidal purple during the course of seed germination and seedling growth. These purple colloidal suspensions showed Au-nanoparticle specific surface plasmon resonance band in absorption spectra. Transmission electron microscopic and powder X-ray diffraction investigations confirmed presence of crystalline Au-nanoparticles in these purple suspensions. Each germinating seed of cowpea released ∼35 nmoles of GAE of phenolics and since phenolics promote generation of Au-nanoparticles, which are less/non toxic compared to Au^3+^, it was contemplated that potential of cowpea to withstand Au^3+^ is linked to phenolics. Of the different components of germinating seed of cowpea tested, seed coat possessed immense power to generate Au-nanoparticles, as it was the key source of phenolics. To establish role of phenolics in generation of Au-nanoparticles (i) seed coat and (ii) the incubation medium in which phenolics were released by germinating seeds, were tested for their efficacy to generate Au-nanoparticles. Interestingly, incubation of either of these components with Au^3+^ triggered increase in generation of Au-nanoparticles with concomitant decrease in phenolics. Accordingly, with increase in concentration of Au^3+^, a proportionate increase in generation of Au-nanoparticles and decrease in phenolics was recorded. In summary, our findings clearly established that cowpea possessed potential to withstand Au^3+^-stress as the phenolics released by seed coat of germinating seeds possess potential to reduce toxic Au^3+^ to form non/less toxic Au-nanoparticles. Our investigations also pave a novel, simple, green and economically viable protocol for generation of Au-nanoparticles.

## Introduction

With the rapid expansion of electronic industry, the demand and cost of gold has increased markedly over past four decades. In general, gold comes into the environment from primary (i.e. ores) and secondary sources such as electronic scrap and waste electroplating solutions [1]–[3]. Au, whose density is 19.32 g cm^−3^, like other heavy metals, has a negative impact on physiology and biochemistry of microorganisms and animal systems including humans [4], [5]. Researchers working with animal systems could trace Au in various organs including ovaries, hypothalamus, liver, adrenals, kidneys, testes, lymph nodes and pituitary glands [6]. In fact, Au has been shown to be transported even over placental barrier into human embryo. In majority of cases, Au was located in lysosomes and whenever the concentration of Au exceeded a certain level, the lysosomal membrane ruptured releasing its contents into the cytosol [6]. In addition, Au also interferes with functioning of energy transducing system (i.e. mitochondria), nuclei and vacuoles [4]. To the best of our knowledge, no significant studies have been carried to investigate the impact of Au on plant growth and development. However, there are reports on accumulation of Au, synthesis and accumulation of Au-nanoparticles in cells of plants exposed to Au salts [7]–[10].

The degree and mechanism of tolerance to heavy metals vary significantly amongst plant species [11]. The basic mechanisms evolved by plants to counter heavy metal tolerance include (i) formation of exogenous non-toxic metal-chelates with organic acids, polyphosphates and siderphores, which restrict metal uptake [12]–[14]; (ii) interaction of toxic metal species with ligands located on cell surface/wall [15]; (iii) active (i.e. energy demanding) efflux of metal ions [16]; and (iv) endogenous chelation/sequestration involving various biomolecules including citrate, oxalate, malate [15], phytochelatins, metallothioneins [17] and phenolic compounds [18], [19]. Many phenolic compounds have been reported to have superior tendency to form stable complexes with most widespread toxic metals such as Ni, Cu, Co and Mn than many organic acids [19]. Phenolics, characterized by at least one aromatic ring (C_6_) bearing one or more hydroxyl groups [20], are a group of low molecular weight secondary metabolites that are known to impart heavy metal stress tolerance either by chelating metal ions or by scavenging heavy metal stress induced reactive oxygen species [18], [19], [21].

Owing to lack of any detailed studies, present investigations were initiated with an aim to evaluate impact of Au^3+^ on growth and development on a leguminous crop, cowpea (*Vigna unguiculata*). Our results showed for the first time that (i) cowpea posesses excellent potential to withstand Au^3+^-stress due to the presence of phenolics; and (ii) phenolics released during the course of seed germination and early seedling growth of cowpea play a vital role in detoxification of Au^3+^ by forming Au-nanoparticles.

## Materials and Methods

Seeds of cowpea [*Vigna unguiculata* (L.) Walp., Fabaceae] were obtained from the local farmers of Haldwani (Uttarakhand, India).

### Impact of Au^3+^

Impact of different concentrations (viz. 0, 0.05, 0.1, 0.25, 0.5 and 1 mM) of Au^3+^ on seed germination and early seedling growth of cowpea was evaluated using HAuCl_4_. After washing with 0.1% cetrimide and distilled water, seeds were surface sterilized with 0.1% mercuric chloride for 2 min, rinsed with sterile distilled water and inoculated in autoclaved bottles containing 75 g of uniform sized glass beads with 20 ml test solution under sterile conditions. These bottles were incubated at 25±2°C under a 16/8 h light/dark cycle at a light intensity of 60 µmol m^−2^ s^−1^. Growth of seedlings was measured in terms of length and fresh weight of root and shoot. Levels of malondialdehyde (MDA) and proline in root and shoot were measured in 4 d old seedlings.

### Estimation of Proline and Malondialdehyde

For determining levels of proline and malondialdehyde (MDA), root and shoot of seedlings were homogenized in 5% TCA and centrifuged at 15,000 xg for 15 min. Proline levels were measured according to Bates et al. [22]. MDA levels were determined following the protocol of Heath and Packer [23]. MDA and proline levels were expressed in nmoles or µmoles g^−1^ fresh weight.

### Estimation of Total Phenolic Content

Total phenolics were measured as per Ainsworth and Gillespie [24] using Folin-Ciocalteau reagent and expressed in terms of nmoles of gallic acid equivalents (GAE).

### Contribution of Different Components of Cowpea to Generate Au-nanoparticles

Different components namely seed coat and cotyledons were excised carefully from 4 d old cowpea seedlings raised in distilled water under sterile conditions. Seed coat, cotyledons and rest of the seedlings (i.e. seedlings devoid of cotyledons+seed coat) were treated with 10 ml of different concentrations of sterile HAuCl_4_ for 24 h to test their potential to generate Au-nanoparticles. Seedlings devoid of cotyledons+seed coat were treated by immersing their roots in HAuCl_4_ solutions. Contol incubation medium (i.e. distilled water), in which seedlings of cowpea were raised for 4 d, was also tested for its efficacy to form Au-nanoparticles by incubating 500 µl of this medium with 10 ml HAuCl_4_.

### Characterization of Au-nanoparticles

UV-Vis spectra of Au^3+^ solutions (i) in which seedlings of cowpea were raised; and (ii) incubated independently with various components of seedlings of cowpea and distilled water in which seedlings were raised as detailed above, were recorded from 190 to 1100 nm using Specord 200 Analytikjena UV-Vis spectrophotometer. For transmission electron microscopic (TEM) studies, 10 µl of colloidal suspension was drop-coated on 200 mesh copper grid with an ultrathin continuous carbon film and allowed to dry in a desiccator at room temperature. Grids were viewed in the transmission electron microscope (Technai G2 T30) at a voltage of 300 kV. The hardware associated with the machine also allowed (i) energy dispersive X-ray (EDX) analysis to measure the elemental composition; and (ii) selected area electron diffraction (SAED) analysis to determine crystalline/amorphous nature, of nanoparticles.

### Powder X-ray Diffraction Studies

For powder X-ray diffraction (PXRD) studies, colloidal suspensions obtained by incubating different components with Au^3+^ solutions were centrifuged. The pellet obtained was re-suspended in distilled water, drop coated on silica surface, dried in desiccator and used for collecting PXRD pattern using Rigaku Rotaflex RAD-B with copper target CuK(α)1 radiation with tube voltage 40 kV and 60 mA in 2 theta (θ) range of 30–80°.

### Stasistical Analysis

All experiments were carried out independently at least seven times. Duncan's multiple range test was used to to determine the level of significance in physiological and biochemical data [25].

## Results and Discussion

### Impact of Au^3+^ on Seedling Growth

**Figure 1:**
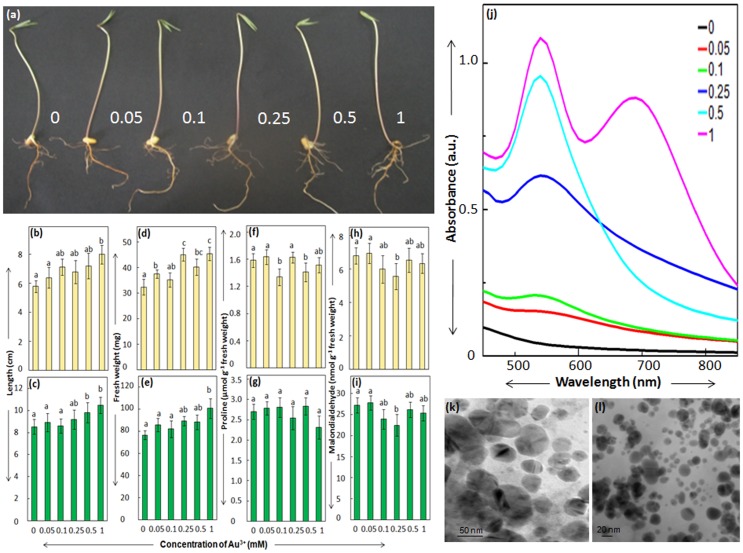
Seedlings of cowpea raised in varying concentrations (mM) of Au^3+^ (a). Length [(b),(c)], fresh weight [(d),(e)], proline [(f),(g)] and MDA levels [(h),(i)] of root (yellow bars) and shoot (green bars) of cowpea seedlings raised in Au^3+^. Absorption spectra (j) and TEM images [(k),(l)] of Au^3+^ solutions in which seedlings were raised. Values represent mean of data collected from seven independent experiments and vertical lines on bars represent standard error [n = 35 for (b), (c), (d) & (e); n = 7 for (f), (g), (h) & (i)]. Values designated by different small letters above bars are significantly different at *P*≤0.05 (Duncan's multiple range test).

While evaluating impact of Au-nanoparticles on seed germination and early seedling growth of cowpea, HAuCl_4_ was used as control. To our surprise, in spite of being a heavy metal ion, Au^3+^ was not associated with any significant alteration in seedling growth (measured in terms of length and fresh weight of root and shoot) of cowpea even when present at a concentration of 1 mM (Figure 1). It is well documented that heavy metal ions such as Cd^+^, Zn^2+^, Co^2+^ and Pb^2+^ inhibit plant growth and development [26]. In general, crop plants exposed to heavy metal stress show enhanced levels of stress markers, proline (an imino acid) and MDA (a cytotoxic byproduct of lipid peroxidation), concomitant with suppression in growth [27], [28]. Heavy metal stress induced enhancement in MDA levels is due to lipid peroxidation by reactive oxygen species (ROS) that are generated due to suppression in electron transport system and/or promotion of Fenton's reaction [29]. Increase in level of proline under heavy metal stress is linked to (i) its synthesis to ensure appropriate recycling of NAD(P)^+^ for cellular metabolism including its role as terminal acceptor of light mediated photosynthetic electron transport [29], [30]; (ii) its role in scavenging ROS [31]; and (iii) protection of enzymes and other macromolecular structures/complexes [32]. Astonishingly, seedlings of cowpea raised in Au^3+^, even at concentration as high as 1 mM, did not show any enhancement in levels of proline and MDA, which is in synchronization with unaltered growth (Figure 1). These findings depicted that cowpea possessesremarkable potential to tolerate Au^3+^ by some unique mechanism.

Unexpectedly, clear pale yellow Au^3+^ solutions in which seedlings of cowpea were raised, turned colloidal purple. Such an alteration in color of Au^3+^ solution (from clear pale yellow to colloidal purple) is due to generation of Au-nanoparticles [33], [34]. Absorption spectra of these colloidal purple suspensions showed distinct peak at ∼550 nm which is well documented to arise due to surface plasmon resonance of Au-nanoparticles [33], [34]. In general, intensity of the purple color and Au-nanoparticle specific absorbance peak of these suspensions increased with increase in concentration of Au^3+^ (Figure 1). Occasionally, an additional peak in infra red region was recorded in absorption spectra of 1 mM Au^3+^ solution in which seedlings of cowpea were raised. At present, it is difficult to presume reasons behind the appearance of this IR peak in only 1 mM Au^3+^ and not other concentrations of Au^3+^ in which seedlings of cowpea were raised. Transmission electron microscopic investigations confirmed presence of distinct crystalline nanoparticles in range of 20–50 nm in these purple colloidal suspensions (Figure 1). It is known that ionic speciation state of metals is more toxic compared to nanoparticle speciation state [35]. Therefore, we hypothesize that the potential of cowpea to withstand Au^3+^-stress is linked to its inbuilt potential to generate Au-nanoparticles.

### Hypothesizing the Involvemenmt of Phenolics in Imparting Au^3+^-Stress Tolerance

Control incubation medium (i.e. distilled water) turned brown during the course of seed germination and early seedling growth. It is known that germinating legume seeds release phenolics [36], which impart brown coloration to the incubation medium. Interestingly, each seedling of cowpea released ∼35 nmoles GAE of phenolics during the course of seed germination and early seedling growth. It is well documented that phenolics such as gallic acid, catechin promote generation of Au-nanoparticles [37], [38]. This prompted us to believe that phenolics released by germinating seeds of cowpea could be responsible for generation of Au-nanoparticles. Owing to electron donating capacity of phenolics [21], we believe that phenolics released in large quantities during seed germination and early seedling growth must have reduced Au^3+^ and promoted formation of Au-nanoparticles. This seems to be an ideal Au^3+^-tolerance mechanism exhibited by cowpea, wherein toxic Au^3+^ is converted to less/non-toxic Au-nanoparticles by phenolics released during seed germination and early seedling growth.

### Identifying the Key Component(s) Involved in Generation of Au-nanoparticles

**Figure 2:**
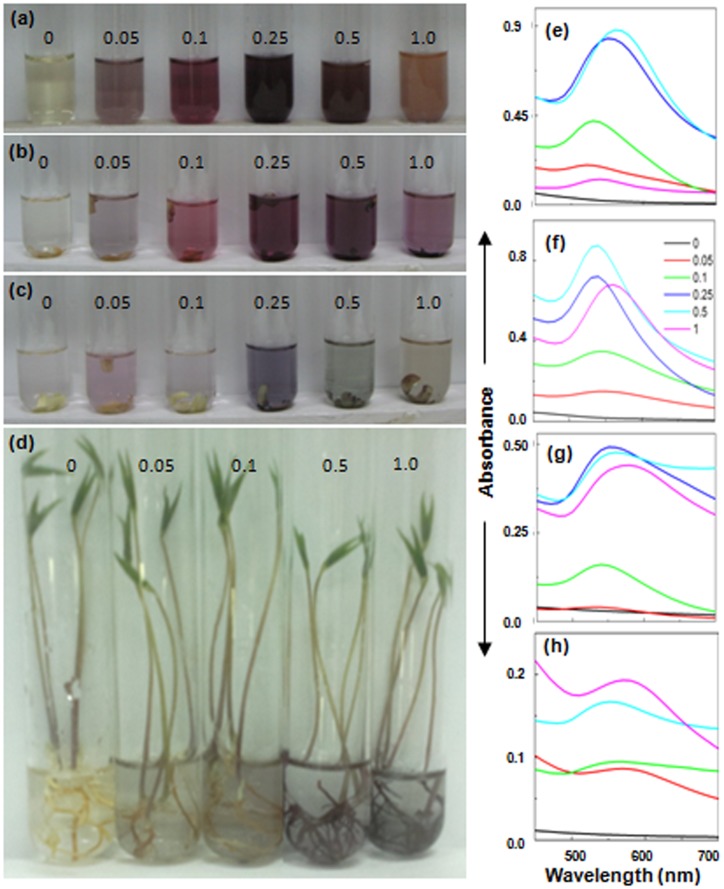
Color (a–d) and absorption spectra (e–f) of Au^3+^ solutions (mM) incubated for 24 h with incubation medium in which cowpea seedlings were raised (a,e); seed coat (b,f); cotyledons (c,g); and roots of intact cowpea seedlings (devoid of cotyledons+seed coat) immersed (d,h).

To identify the key component(s) responsible for formation of Au-nanoparticles and imparting Au^3+^-stress tolerance to cowpea seedlings, four different components namely (i) control incubation medium (i.e. distilled water) in which seedlings were raised; (ii) seed coat; (iii) cotyledons; and (iv) seedlings devoid of cotyledons+seed coat, were tested for their efficacy to generate Au-nanoparticles by independently incubating them in different levels of Au^3+^. As anticipated, brown colored incubation medium possessed abundant potential to turn pale yellow Au^3+^ solutions purple indicating the generation of Au-nanoparticles (Figure 2). Of the three components of 4 d old seedlings (viz. seed coat, cotyledons and seedlings devoid of cotyledons+seed coat), seed coat possessed maximum potential to turn pale yellow Au^3+^ solutions purple (Figure 2). Accordingly, Au-nanoparticle specific plasmon resonance band in absorprion spectra of purple suspensions formed by (i) incubation medium in which seedlings were raised and (ii) seed coat, was more intense compared to those formed by cotyledons and seedlings devoid of cotyledons+seed coat.

**Figure 3:**
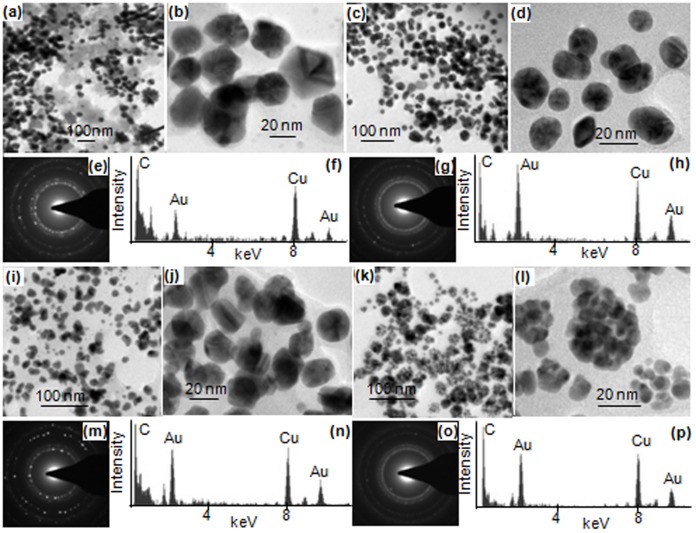
TEM images (a–d,i–l), SAED pattern (e,g,m,o) and EDX spectra (f,h,n,p) of Au-nanoparticles in Au^3+^ solutions incubated with incubation medium in which cowpea seedlings were raised (a,b,e,f); seed coat (c,d,g,h); cotyledons (i,j,m,n); and the roots of intact cowpea seedlings (devoid of cotyledons+seed coat) immersed (k,l,o,p).

**Figure 4:**
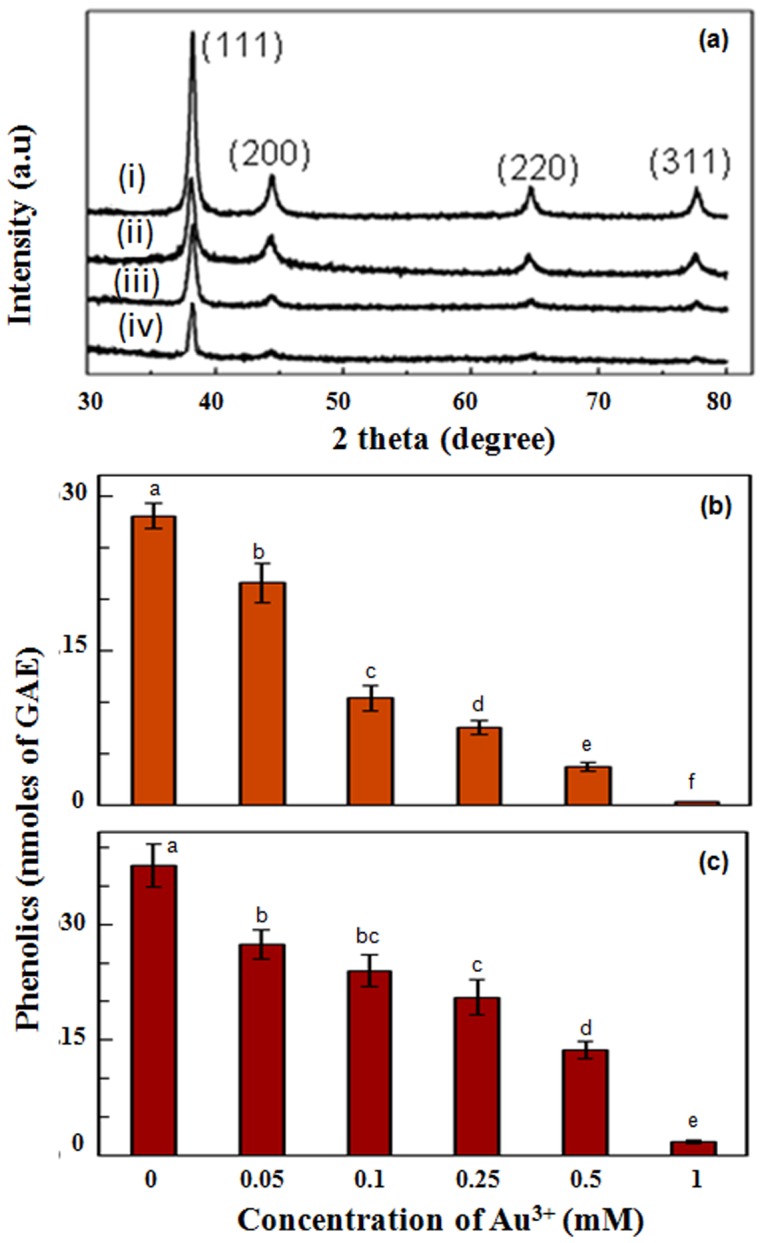
PXRD pattern of Au-nanoparticles (a) in Au^3+^ solutions incubated with incubation medium in which cowpea seedlings were raised (i); seed coat (ii); cotyledons (iii); and roots of intact cowpea seedlings (devoid of cotyledons+seed coat) immersed (iv); Phenolic content [(b),(c)] in Au^3+^ solutions (mM) incubated with incubation medium (b); and seed coat (c). Values represent mean of data collected from seven independent experiments and vertical lines on bars represent standard error (n = 7). Values designated by different small letters above bars are significantly different at *P*≤0.05 (Duncan's multiple range test).

Transmission electron microscopic investigations confirmed presence of nanoparticles in purple colloidal suspensions formed independently by all four components when incubated with Au^3+^ solutions. However, the size and morphology of nanoparticles varied depending on the component. Seed coat and incubation medium generated nanoparticles in range of 10–30 nm, while cotyledons and seedlings devoid of cotyledons+seed coat generated nanoparticles in the range of 10–25 and 5–10 nm, repectively (Figure 3). These results indicated that the mechanism of generation of nanoparticles in the former two cases is similar, but, vary distinctly from the latter two cases. However, irrespective of the component responsible for generation of Au-nanoparticles, EDX spectra showed two prominent peaks of Au confirming that these nanoparticles were composed of Au (Figure 3). The prominent peaks of Cu and C seen in these EDX spectra arose from carbon coated copper grids, on which samples were loaded. Irrespective of the component responsible for generation of Au-nanoparticles, the nanoparticles were crystalline as revealed by SAED pattern (Figure 3). Similarly, PXRD analysis showed that Au-nanoparticles formed in all cases were crystalline and had face centered cubic structure corresponding to (111), (200), (220), and (311) gold crystalline facets which matched with the JCPDS (Joint Committee on Powder Diffraction Studies) File No. 04–0784 (Figure 4).

### Establishing Role of Phenolics in Imparting Au^3+^-stress Tolerance

Interestingly, as evident from figure 2d, seedlings devoid of cotyledons+seed coat showed significant suppression in growth upon exposure to Au^3+^ solutions at concentration above 0.1 mM. This is in contrast to the unperturbed growth response shown by seedlings raised in presence of different levels of Au^3+^ (Figure 1) and seedlings with cotyledons+seed coat incubated in different levels of Au^3+^ (data not shown). Potential of cowpea seedlings with seed coat to generate 5–6 fold higher level of Au-nanoparticles compared to the seedlings devoid of seed coat is evident from the intensity of Au-nanoparticle specific absorption peak of purple colloidal suspensions formed by them (Figures 1 and 2). This clearly established that seed coat, which is the key source of phenolics play a pivotal role in imparting Au^3+^ tolerance to cowpea.

Close correlation between potential of seed coat to release large quantity of phenolics with its potential to generate large proportion of Au-nanoparticles, as evident from figure 2, strengthens our hypothesis that phenolics released during seed germination play a vital role in imparting Au^3+^-stress tolerance through formation of Au-nanopartoicles. This hypothesis was further strengthened by the potential of control incubation medium (distilled water in which seeds were germinated and seedlings raised) that contained large quantity of phenolics to generate large proportion of Au-nanoparticles (Figure 2).

To establish direct role of phenolics in generating Au-nanoparticles, the level of phenolics in Au^3+^ solutions incubated with seed coat and incubation medium (in which seeds were germinated and seedlings raised) were determined. As evident from figure 4, level of phenolics decreased progressively with increase in concentration of Au^3+^. This clearly established that phenolics played a critical role in reduction of Au^3+^ and generation of Au-nanoparticles. As stated earlier, phenolics possess potential to donate electrons to metal ions such as Au^3+^ and promote synthesis of Au-nanoparticles.

In nutshell, our results convincingly demonstrated that seed coat, being key source of phenolics, is the most powerful component of developing seedling responsible for generation of Au-nanoparticles. In light of above elaborated experimental evidences, we believe that seed coat plays most vital role in imparting Au^3+^-stress tolerance to cowpea. Earlier researchers had reported that seed coat plays important role in chemical protection from oxidative damage as it possesses phenolics which act as antioxidants [39], [40]. Although, we do not rule out the possible role of phenolics in acting as antioxidants as has been established by these researchers, our results convincingly demonstrated for the first time that phenolics released by seed coat impart Au^3+^-stress tolerance by rapidly converting toxic ionic speciation state into less/non-toxic nanoparticle speciation state.

## Conclusions

Our above findings demonstrated for the first time that cowpea has substantial potential to withstand Au^3+^-stress during seed germination and early seedling growth. Our results established that (i) the potential of cowpea to withstand Au^3+^-stress is linked to phenolics; (ii) seed coat is the key source of phenolics during seed germination and early seedling growth and hence is important for imparting Au^3+^-stress tolerance; and (iii) phenolics impart Au^3+^-stress tolerance by converting toxic ionic speciation state of Au to less/non-toxic nanoparticle speciation state. Owing to the proven role of phenolics in imparting metal ion stresss tolerance, we believe that it is important to understand molecular and genetic basis of modulating the synthesis of phenolics for improving tolerance of agricultural/forestry plant species against metal ion stress. Our findings also furnish a novel, simple, green and economically viable protocol for using seed/seed coat of legume seeds for synthesis of metal nanoparticles.
